# Human RNA Polymerase II-Association Factor 1 (hPaf1/PD2) Regulates Histone Methylation and Chromatin Remodeling in Pancreatic Cancer

**DOI:** 10.1371/journal.pone.0026926

**Published:** 2011-10-27

**Authors:** Parama Dey, Moorthy P. Ponnusamy, Shonali Deb, Surinder K. Batra

**Affiliations:** 1 Department of Biochemistry and Molecular Biology, University of Nebraska Medical Center, Omaha, Nebraska, United States of America; 2 Eppley Institute for Research in Cancer and Allied Diseases, University of Nebraska Medical Center, Omaha, Nebraska, United States of America; Texas A&M University, United States of America

## Abstract

Change in gene expression associated with pancreatic cancer could be attributed to the variation in histone posttranslational modifications leading to subsequent remodeling of the chromatin template during transcription. However, the interconnected network of molecules involved in regulating such processes remains elusive. hPaf1/PD2, a subunit of the human PAF-complex, involved in the regulation of transcriptional elongation has oncogenic potential. Our study explores the possibility that regulation of histone methylation by hPaf1 can contribute towards alteration in gene expression by nucleosomal rearrangement. Here, we show that knockdown of hPaf1/PD2 leads to decreased di- and tri-methylation at histone H3 lysine 4 residues in pancreatic cancer cells. Interestingly, hPaf1/PD2 colocalizes with MLL1 (Mixed Lineage Leukemia 1), a histone methyltransferase that methylates H3K4 residues. Also, a reduction in hPaf1 level resulted in reduced MLL1 expression and a corresponding decrease in the level of CHD1 (Chromohelicase DNA-binding protein 1), an ATPase dependent chromatin remodeling enzyme that specifically binds to H3K4 di and trimethyl marks. hPaf1/PD2 was also found to interact and colocalize with CHD1 in both cytoplasmic and nuclear extracts of pancreatic cancer cells. Further, reduced level of CHD1 localization in the nucleus in hPaf1/PD2 Knockdown cells could be rescued by ectopic expression of hPaf1/PD2. Micrococcal nuclease digestion showed an altered chromatin structure in hPaf1/PD2-KD cells. Overall, our results suggest that hPaf1/PD2 in association with MLL1 regulates methylation of H3K4 residues, as well as interacts and regulates nuclear shuttling of chromatin remodeling protein CHD1, facilitating its function in pancreatic cancer cells.

## Introduction

Post-translational modifications of basic histone proteins, such as phosphorylation, methylation, acetylation, ubiquitylation or sumoylation, play a major role in regulating gene expression. One way by which such histone modifications function is by recruitment of downstream effector proteins which perform specific independent functions on the chromatin template. These include the chromatin remodeling proteins which “read” the modified histone marks and use ATP energy to alter the position of nucleosomes on the DNA. As a result, the chromatin template is either blocked or made accessible to the transcription machinery, hence regulating downstream gene expression. Histone methylation, specifically the histone H3 lysine 4 residue mono-, di- and trimethylation, is mostly associated with 5′ regions of actively transcribed genes [1]. However, a recent study shows that in regions of DNA damage, ING proteins recruit HDAC complexes to silence transcription of cell proliferation genes through recognition of trimethylated H3K4 residues by their PHD domains [2]. This is the first report linking K4 trimethylation to actively repressed genes as well.

In mammals, the methylation at the H3K4 residue is carried out by the histone methyltransferase MLL1, a SET domain containing protein which remains in a complex with other subunit proteins WDR5, RbBP5 and ASH2 [3]. The yeast homologue of MLL1, Set1, is a part of a macromolecular complex called COMPASS (complex of proteins associated with Set1) that interacts with yeast PAF complex for histone methylation [4]. It has been postulated that some conserved genes, from Drosophila to humans, bear this methylation mark exclusively and the expression of these genes is regulated by Pol II general elongation factors [1]. The H3K4 histone methylation marks can be further recognized by specific proteins, resulting in recruitment and subsequent enzymatic or physiological activity at the site of recruitment. CHD1 (Chromodomain Helicase DNA binding protein 1) is a protein belonging to the family of ATPase dependent chromatin remodeling factors that specifically associate with dimethylated and trimethylated H3K4 [5], [6]. The human CHD1 binds to the methylated histone H3K4 residue through both of its tandem chromodomains, whereas the yeast Chd1 fails to bind methylated H3K4 residues [7].

Cancer development and progression is attributed to dysregulated gene expression which might often correlate to either faulty epigenetic activation or silencing of genes [8]. Altered histone modification patterns cause mistargeting of chromatin remodeling enzymes that might lead to uncontrolled gene expression and hence cause normal cells to be transformed into cancer cells. Pancreatic cancer is the fourth leading cause of cancer with a very poor prognosis and five year survival rate of less than 5%. Like other forms of malignancy, pancreatic cancer is also correlated to epigenetic alterations such as DNA methylation and histone modifications, leading to an altered gene expression [9].

PD2 is the human homologue of the yeast RNA polymerase II-associated factor 1 (yPaf1) which constitutes the core subunit of the human RNA Polymerase II associated factor (hPAF) complex. Similar to its yeast counterpart, the hPAF complex is comprised of four other subunits, namely hLeo1, hCtr9, hSki8 and parafibromin [10]–[12]. The major functions of the hPAF complex involves transcriptional elongation, mRNA processing and maturation, similar to that of the yeast PAF complex [12]. Chromatin modification and remodeling are also some of the characteristics of transcription factors. Evidently, a host of histone modifications have been linked to the PAF complex and transcription elongation as well [4], [13]. Both the yeast and the human PAF complex are found to be necessary for Rad6/Bre1-dependent ubiquitylation of histone H2B [14]. The monoubiquitylation of H2B by hPAF removes the nucleosomal barrier, which is a prerequisite for efficient transcription elongation on chromatin by RNA Pol II [14]. The presence of ubiquitylated H2B further triggers H3K4 di and tri-methylation. In yeast, the Paf1C function in H3K4-Me3 formation is conferred by the Rtf1 subunit by recruiting the SET1 histone methyltransferase complex [15], which is homologous to the human MLL complex [16]. Yeast studies also show that the Paf1 complex is required for recruitment of the COMPASS methyltransferase complex to RNA polymerase II through subunit interaction. In human cells, hCdc73 appears to be required for full levels of H3K36-Me3 [17], but not H3K4-Me3, whereas in yeast Cdc73 is required for both modifications [18]. Therefore, the role of PAF complex in transcription elongation has been closely correlated to histone modifications.

Besides its role in transcription regulation, the human PAF complex subunits have also been linked to a malignant phenotype. Parafibromin (hCdc73) is found to be mutated in parathyroid tumors and amplified in liver and breast carcinoma. Leo1, present at the 15q21 locus, is amplified in colorectal cancer and malignant fibrous histiocytoma of the bone, whereas the *Ctr9* gene locus is found to be deleted in pancreatic cancer. The hPaf1 subunit of the human PAF complex, also known as PD2, is amplified and overexpressed in pancreatic cancer and its possible role in tumorigenesis is indicated by the induction of a transformed phenotype on overexpression [19]. In this study, we identify a role of hPaf1/PD2 in regulating histone methylation at the H3K4 residue in pancreatic cancer cells by interaction with histone methyltransferase MLL1. It was also found to regulate the expression of the chromatin remodeling factor, CHD1 that specifically binds to di and trimethylated H3K4 and facilitate its nuclear import, probably through direct interactions. Further, micrococcal nuclease digestion assays show that knockdown of PD2 leads to altered nucleosomal positioning in pancreatic cancer cells.

## Materials and Methods

### Cell culture

Human pancreatic cancer cell lines Panc1 and MiaPaca were obtained from ATCC. The cells were culture in DMEM media (Sigma) supplemented with 10% fetal bovine serum (Sigma) and 1% pencillin-streptamycin solution (Sigma). Culture medium was changed every 2 days and cells were subcultured by trypsin-EDTA treatment.

### RNA Isolation and QRT-PCR

Total cellular RNA was extracted from Panc1 cells using the RNAeasy kit (Qiagen) and processed for reverse transcription. The initial PCR activation step was at 94°C for four minutes, followed by the denaturation step at 94°C for one minute, primer-annealing step at 58°C for 30 seconds, extension step at 72°C for one minute, and the final extension step at 72°C for ten minutes. PCR reaction products were then separated by electrophoresis using a 2% agarose gel. Gels were stained using 0.5 µg/ml of ethidium bromide, illuminated with UV light. Total cell RNA was reverse-transcribed and assayed by quantitative real-time PCR using SYBR Green incorporation. The expression of all genes was normalized to that of the internal control β-actin and expressed relative to the indicated reference sample (average ± S.D. of triplicate reactions). The expressions of lineage specific genes were compared between the scrambled and PD2 knockdown Panc1 cells by the two-tailed Student's t-test. A p-value of <0.05 was considered statistically significant.

### RNA Interference

The region of Paf1/PD2 was targeted with specific siRNA (sequence 5′- AACAGGUUCGUCCAGUACAAA-3)′. Synthetic sense and antisense oligonucleotides (Dharmacon, Lafayette, CO) were annealed in 100 mM potassium acetate, 30 mM HEPES-KOH (pH 7.4), and 2 mM magnesium acetate for one minute at 90°C and one hour at 37°C, and frozen. Oligonucleotides were transfected into cells with *Trans*IT-TKO (Mirus, Madison, WI) in accordance with the supplier's recommendations.

### Immunoblot Assay

Panc1 and MiaPaCa cell lines were processed for protein extraction and Western blotting using standard procedures. Briefly, the cells were washed twice in PBS and lysed in RIPA buffer (100 mM Tris, 5 mM EDTA, 5% NP40;pH 8.0) containing protease inhibitors (1 mM phenyl-methyl sulphonyl fluoride, 1 µg/ml aprotinin, 1 µg/ml leupeptin) and kept at 4°C and supernatants were collected. Twenty µg of protein lysates was resolved in 10% SDS-PAGE. Resolved proteins were transferred on to the PVDF membrane. After quick washing in PBST (Phosphate buffered saline and 0.1% Tween 20), the membranes were blocked in 5% nonfat dry milk in PBS for at least 2 h and then incubated with primary antibodies (anti-Paf1/PD2, anti-Leo1, anti-Cdc, anti-Ski8, anti- Oct3/4, anti-SOX2, anti-β-actin) (diluted in 3% BSA in PBS) overnight at 4°C. The membrane was then washed (3×10 min) in PBST at room temperature and probed with 1∶2000 diluted horseradish peroxidase-conjugated anti-mouse or anti-rabbit secondary antibodies for 1 h at room temperature and washed 5×10 min with PBST. The signal was detected with an ECL chemiluminescence kit (Amersham Bioscience, UK). All the western blots were quantified using the ChemiImager 4400 software. The graphs generated show error bars representing the standard errors as calculated from three independent experimental replicates. The difference in each protein level between scrambled and PD2 knockdown cells are analyzed by Students t-test with a p-value of less than 0.05 being considered statistically significant.

### Confocal Microscopy

Cells were plated onto sterile round cover slips (CIR 18-1 Fisher brand 12-545-10) and grown in 12-well plates for 24 hrs. Cells were fixed in ice cold methanol (pre-chilled to −20°C) and permeabilized with 0.1% Triton X-100 in PBS. Then, the cells were washed in PBS and blocked using 10% goat serum for 30 mins. After blocking, it was incubated with primary (PD2 mouse monoclonal, CHD1 rabbit polyclonal) antibodies for one hour and fluorescent tagged secondary antibodies (FITC goat anti-mouse, Texas-Red goat anti-rabbit) for 30 mins at room temperature. Antibodies were diluted in 5% goat serum. Finally, following secondary antibody incubation, the cells were washed with TBST three times and cover slips were mounted with vectashield containing DAPI (VECTOR).

### Isolation of cytoplasmic and nuclear extract

The protocol for cytoplasmic and nuclear extract preparation was adapted from previously published procedures. Cells were rinsed twice in ice cold PBS and incubated on ice for 30 minutes in hypotonic cytoplasmic extraction buffer (10 mM Hepes, pH 7.4, 10 mM KCl, 0.2% NP-40, 0.1 mM EDTA, 10% glycerol, 1.5 mM MgCl_2_, 1 mM DTT, 1 mM PMSF, 5 mM Na_3_VO_4_, 5 mM NaF), supplemented with complete protease inhibitor cocktail [Roche]. Cell disruption was accomplished by several passages through a 25G needle. Nuclei were collected by centrifugation at 16,000 g, and the supernatant further centrifuged at 16,000 g to yield the final cytoplasmic extract. Nuclear pellets were washed twice with ice cold PBS followed by incubation in hypertonic nuclear extraction buffer (20 mM Hepes, [pH 7.6], 420 mM NaCl, 1 mM EDTA, 20% glycerol, 1.5 mM MgCl_2_, 1 mM DTT, 1 mM PMSF, 5 mM Na_3_VO_4_, 5 mM NaF), supplemented with complete protease inhibitor cocktail [Roche]. The solution was further sonicated at 60% amplitude twice for 10 seconds each. Insoluble materials were precipitated by centrifugation. The supernatant was collected and used as nuclear extract.

### Immunoprecipitation Analysis

Equal amounts of Protein A+ G-Sepharose beads (Oncogene Research, Boston, MA) were incubated overnight with anti-PD2 (rabbit polyclonal) and anti-CHD1 (rabbit polyclonal) antibodies in a 1 ml total volume. It was then washed twice to remove the unbound antibody and 1.5 mg of protein lysate was added to the bead-antibody mix and incubated on a rotating platform for 5 h at 4°C. Following the incubation period, the mixtures were washed five times with lysis buffer. The immunoprecipitates or total cell lysates were electrophoretically resolved on SDS-PAGE (10%). Resolved proteins were transferred on to the PVDF membrane. After quick washing in PBST (Phosphate buffered saline and 0.1% Tween 20), the membranes were blocked in 5% nonfat dry milk in PBS for at least 2 h and then incubated with primary antibodies (anti- Paf1/PD2, Oct3/4 and RNA Pol II). The immunoblots were washed five times (5×10 min), incubated 1 h with horseradish peroxidase-conjugated secondary antibodies, washed five times (5×10 min), reacted with enhanced chemiluminescence ECL reagent (Amarsham Bioscience, Buckinghamshire, UK), and exposed to X-ray film to detect the signal.

### Micrococcal Nuclease Assay

The micrococcal nuclease assay was carried out as mentioned earlier [20] with some modifications. Fifty×10^6^ cells were pelleted at 2000 RPM at 4°C for 10 minutes, followed by washing with 10 ml of ice-cold PBS and again pelleted as before. The cells were then resuspended in 5 ml of ice-cold NP-40 lysis buffer (10 mM tris-HCl (pH 7.4), 10 mM NaCl, 3 mM MgCl_2_, 0.5% Nonidet P-40, 0.15 mM spermine and 0.5 mM spermidine), incubated for 5 mins on ice and the nuclei were repelleted. The nuclei was washed with 2.5 ml of MNase digestion buffer (10 mM Tris-HCl, 15 mM NaCl, 60 mM KCl, 0.15 mM spermine and 0.5 mM spermidine) and after centrifugation further resuspended in 1 ml of MNase digestion buffer containing 2 mM CaCl_2_. From the resuspended solutions, 100 µl aliquots were taken and incubated with increasing amounts of the Micrococcal Nuclease enzyme (0, 80 and 100 units) for 5 and 10 minutes. The reaction was stopped by adding 80 µl of MNase digestion buffer, 20 µl of MNase stop buffer (100 mM EDTA, 10 mM EGTA), 3 µl of Proteinase K (25 mg/ml) and 10 µl of 20% SDS and incubated overnight at 37°C. The following day, the samples are extracted with 200 µl of phenol-chloroform solution. The samples were spun and an aqueous layer was collected. Two µl of RNAse A (10 mg/ml) was added to the solution and incubated at 37°C for 2 hours and again phenol-chloroform extraction was performed. Further, the DNA was precipitated by adding 100% ethanol and after centrifugation, the DNA pellet was resuspended in DNA hydration buffer or water. Five µg of the isolated DNA was resolved in a 1.4% agarose gel and visualized by ethidium bromide staining. ImagJ software was used for densitometry analysis of the DNA banding patterns in control, Scrambled and PD2 KD PC cells.

## Results

### hPaf1/PD2 affects other PAF complex subunits in PC cells

**Figure 1:**
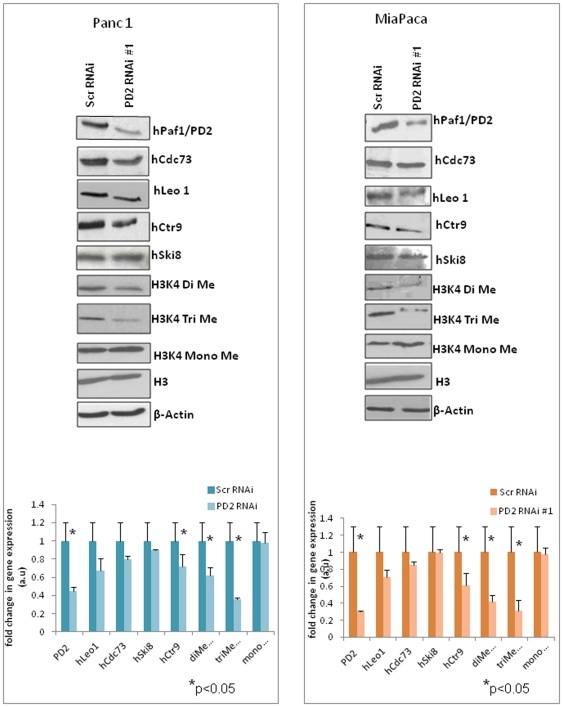
Expression of human PAF complex molecules in scrambled and PD2 RNAi Panc1 and MiaPaCa cells. Panc1 and MiaPaCa pancreatic cancer cells were transfected with scrambled and PD2 specific siRNA oligos and protein lysates were collected at 72 hours post-transfection. Western blot analysis of the cell lysates using corresponding antibodies showed the expression of hPaf1/PD2 and other PAF complex molecules (parafibromin, hLeo1, hSki8 and hCtr9) in scrambled and Paf1/PD2 RNAi treated Panc1 and MiaPaCa cells. The expression of histone 3 Lysine 4 residue mono, di and tri-methylation marks showed a reduced level in hPaf1/PD2 knockdown Panc1 and MiaPaCa cells compared to scrambled RNAi treated cells. The total Histone H3 level is the same in both scrambled and PD2 knocked down cells. β-actin served as a loading control. Quantification of the western blots for each cell line is provided below the immunoblot figures with corresponding error bars. * represents significant change (p<0.05) in protein level between scrambled and PD2 knockdown cells.

The human PAF complex consists of five subunits, similar to its yeast counterpart, namely hPaf1, parafibromin (hCdc73), hCtr9, hLeo1 and the hSki8 subunit, which is also a part of the human SKI complex. Earlier reports have shown that the subunits of the human PAF complex demonstrate coordinated expression where knockdown of either hCtr9 or the hSki8 subunit led to a reduction in levels of the other subunits such as hPaf1 and hLeo1 [12]. This is consistent with the observation in yeast, where deletion of individual subunits leads to various degrees of reduction in the protein levels of other subunits [21]. Interestingly, recent data from our lab has shown that, the PAF complex subunits function independently in mouse embryonic stem cells, such that knockdown of the Paf1 subunit does not affect the level of the other subunits [22]. To analyze the functional importance of hPaf1, the major subunit of the hPAF complex, in pancreatic cancer, we transiently knocked down hPaf1/PD2 in the pancreatic cancer cell lines Panc1 and MiaPaCa. In corroboration with earlier reports, we found that hPaf1/PD2 has a coordinated expression with the other subunits of the hPAF complex. Western Blot analysis of hCdc73 (parafibromin), hLeo1, hSki8 and hCtr9 proteins and subsequent quantification showed decreased expression levels of the hLeo1,hCdc73 and hCtr9 subunit in the hPaf1 knockdown Panc1 and MiaPaCa cells compared to the scrambled siRNA treated cells (**Fig. 1**) although not statistically significant. (p>0.05) However there was no observable change in the protein level of hSki8 subunit upon downregulation of the hPaf1 subunit. We further confirmed the results by using a different pool of siRNAs (Santa Cruz, CA) directed against PD2 and observed similar effects (**Fig. S3**). These results show that knockdown of one subunit of the hPAF complex affects the expression of the other members in order to maintain a general stoichiometric ratio of the complex in pancreatic cancer cells. Further, it also emphasizes the differential expression and function of individual subunits of the PAF complex in different carcinomas.

### hPaf1/PD2 influences histone methylation in PC cells

The yeast PAF complex has a well established role in histone modifications such as ubiquitylation and methylation [4], [23]–[25]. It is known to mediate the interaction among the Rad6-Bre1 complex, COMPASS histone methyltransferase, and RNA Pol II, leading to the ubiquitination of histone H2B at lysine 123 residue, which is a subsequent trigger for methylation at the lysine 4 residue of Histone H3 [24]. An earlier study has shown that RNAi against hCtr9 or hSki8 led to a reduction in the cellular level of histone H3K4 mono and trimethylation marks but not dimethylation marks in Hela cells, indicating a possible role of the human PAF complex in histone methylation as well [12]. We found that transient knockdown of hPaf1/PD2 using specific RNAi in pancreatic cancer cells affects histone methylation at the H3K4 residue (**Fig. 1**). Western Blot analysis of mono, di and trimethylation marks at the histone H3 lysine4 residue showed a significant reduction (*p<0.05) in di- and trimethylated H3K4 levels in PD2 knockdown Panc1 and MiaPaCa cells as compared to scrambled RNAi treated cells. However, there was not much variation in the H3K4 monomethylation levels due to PD2 depletion in pancreatic cancer cells. The experiments were repeated with a different pool of PD2 specific siRNAs in both cell lines (**Fig. S3**). We again observed downregulation of histone di- and trimethylation marks as before with little or no variation in histone monomethyl levels.

### hPaf1/PD2 regulates histone methyltransferase MLL1 protein level

**Figure 2:**
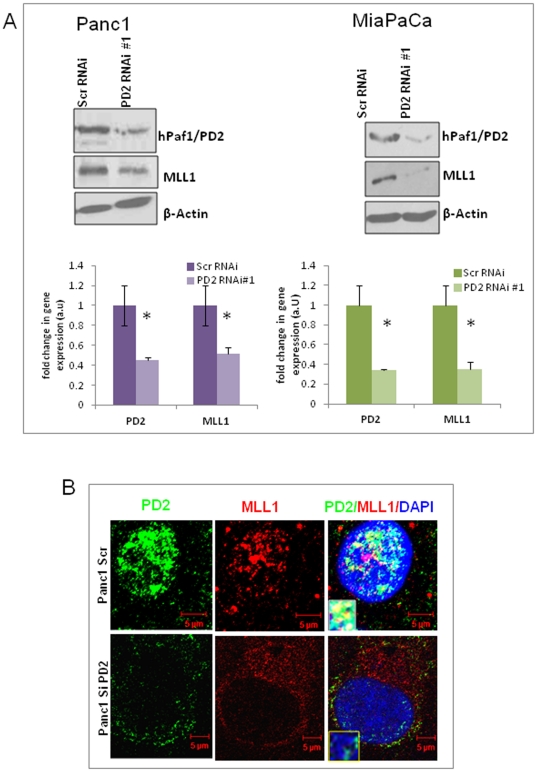
Expression of histone methyltransferase MLL1 in hPaf1/PD2 knockdown PC cells. (**A**) Panc1 and MiaPaCa pancreatic cancer cells were transfected with scrambled and PD2 RNAi oligos and lysates were collected at 72 hours post transfection. Western Blot analysis using specific antibodies shows a decrease in MLL1 histone methyltransferase protein expression in knockdown cells compared to scrambled cells. β-actin served as a loading control. Quantification data of the western blot is provided below the corresponding immunoblot results for respective cell lines. The error bars indicated represent standard error calculated from three independent experiments. * represents significant change (p<0.05) in protein level between scrambled and PD2 knockdown cells. (**B**) Confocal microscope images illustrating PD2 and MLL1 localization in the nucleus of Panc1 cells. MLL1 colocalizes with PD2 in discrete regions within the nucleus (stained with DAPI) of Panc1 cells. The inset represents a magnified image of the colocalization spots of PD2 and MLL1.

Based on the observation that hPaf1/PD2 regulates histone di and tri-methylation at the H3K4 residue, we wanted to investigate the effect of hPaf1 knockdown on histone methyltransferases. The MLL1 protein belonging to the SET1 family of lysine methyltransferases is known to specifically regulate di-and trimethylation at histone H3 lysine 4 residues. Therefore, we analyzed the effect of hPaf1/PD2 knockdown on MLL1 protein expression using two different sets of PD2 siRNAs in Panc1 and MiaPaCa pancreatic cancer cell lines. Western blot analysis revealed that a decrease in hPaf1/PD2 level in pancreatic cancer cells leads to a significant decrease (*p<0.05) in MLL1 protein level (**Fig. 2A** **, S3**). The graphs provided below the immunoblotting results represent quantification of the western blots for each cell line. We further looked at PD2 and MLL1 proteins in pancreatic cancer cells by confocal microscopy and found that the two proteins colocalize in the nucleus, thereby suggesting a possible interaction between them (**Fig. 2B**). However, the amount of colocalization of the two proteins was relatively low, which indicates a possibly weak and/or transient interaction. These results were in corroboration with earlier studies which showed that both the yeast and human PAF complexes interact with the corresponding homologue of MLL or Set1 protein [4], [26], [27]. Therefore, our results suggest that hPaf1/PD2 regulates, and possibly interacts with MLL1, to control H3K4 methylation in pancreatic cancer cells.

### Knockdown of hPaf1/PD2 decreases CHD1 level in pancreatic cancer cells

**Figure 3:**
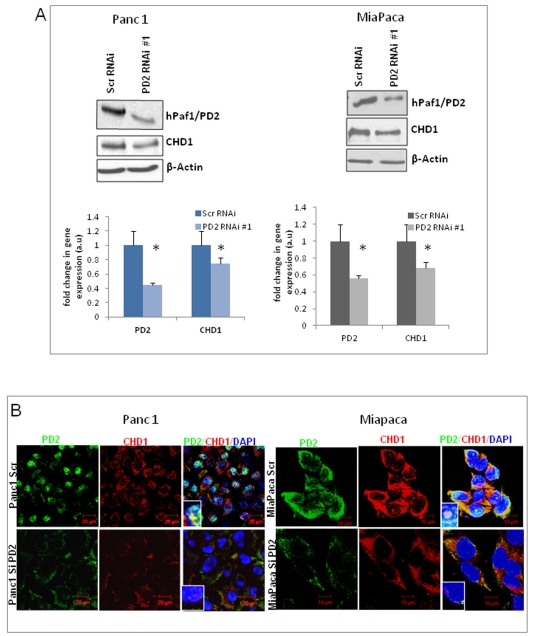
Downregulation of hPaf1/PD2 reduces the CHD1 protein level in PC cells. (**A**) Western blot analysis shows a decreased level of the CHD1 protein in PC cells upon RNAi mediated hPaf1/PD2 inhibition. β-actin served as a loading control. The graph below each western blot figure represents the corresponding quantification results for individual cell lines. (**B**) Immunofluorescence analysis showing a reduced hPaf1/PD2 level (green) along with a corresponding reduction in CHD1 level (red) in PD2 siRNA treated PC cells compared to scrambled siRNA treated cells.

The regulation of histone methylation by the hPaf1/PD2 subunit of the hPAF complex raises the possibility that it might affect various other proteins, such as chromatin remodeling factors, which interact with modified histones at sites of transcription. A putative chromatin remodeling complex, CHD-1 (Chromodomain Helicase DNA-binding protein 1), was identified adventitiously as a mammalian DNA-binding protein, which contains three signature sequence motifs: the 52-amino acid chromodomain found in the heterochromatin protein HP-1 and the homeotic repressor Polycomb, the SNF2/SWI2 ATPase domain, and motifs characteristic of minor-groove DNA binding proteins [28], [29]. Interestingly, the CHD1 chromatin remodeling protein is known to specifically bind to the methylated lysine 4 residue of histone H3, which is a hallmark of active transcription. It is postulated that the human CHD1 chromodomains are responsible for recognition and interaction with the histone methylation marks [30]. Given that hPaf1/PD2 regulated histone methylation at the H3K4 residue, we investigated CHD1 regulation by hPaf1 by transient knockdown of hPaf1/PD2 in pancreatic cancer cells and analyzed the variation in levels of the CHD1 protein. Western blot analysis along with corresponding quantification data provided below the immunoblotting figures (**Fig. 3A**) shows that knockdown of hPaf1/PD2 in pancreatic cancer cells leads to a decrease in the level of CHD1 compared to the scrambled control cells. Downregulation of CHD1 as an effect of PD2 knockdown was further confirmed in the same cell lines using a different siRNA pool against PD2 (Fig. S3). In order to validate this effect, immunofluorescence microscopy was used. Consistent with the biochemical data, the immunofluorescence analysis showed that the CHD1 level is decreased in hPaf1/PD2 knockdown Panc1 and MiaPaCa cells compared to the scrambled MiaPaCa or Panc1 cells (**Fig. 3B**). Quantitative real-time PCR analysis further shows that along with PD2 mRNA, there is also a decrease in CHD1 mRNA level in PD2 siRNA treated Panc1 cells, indicating that hPaf1 regulates CHD1 expression both at the transcription and translational levels (**Fig. S1A**). Concurrently, overexpression of PD2 in HPAF/CD18 pancreatic cancer cells also upregulates CHD1 expression (**Fig. S1B**). These results indicate that hPaf1/PD2 regulates the expression of the chromatin remodeling protein CHD1, suggesting its possible role in the rearrangement of the chromatin structure during transcription elongation in pancreatic cancer cells.

### hPaf1/PD2 interacts with CHD1 in both the cytoplasm and the nucleus of pancreatic cancer cells

**Figure 4:**
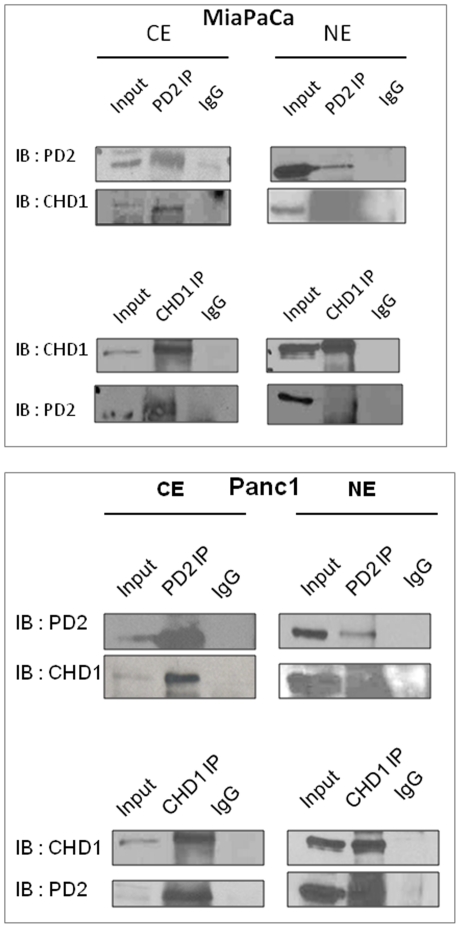
Interaction of hPaf1/PD2 with CHD1 in pancreatic cancer cells. (**A**) Cytoplasmic and nuclear extract fractionation was performed in Panc1 and MiaPaCa pancreatic cancer cells followed by reciprocal co-immunoprecipitation of endogenous proteins in the cell fractions. Western blot analysis shows that hPaf1/PD2 interacts with CHD1 in both cytoplasmic and nuclear extracts of Panc1 and MiaPaCa pancreatic cancer cells. Anti-PD2 immunoprecipitates from extracts of Panc1 and MiaPaCa cells were immunoblotted with an anti-CHD1 antibody. Similarly, anti-CHD1 precipitates were blotted with anti-PD2 antibody. Rabbit IgG was used as a control.

**Figure 5:**
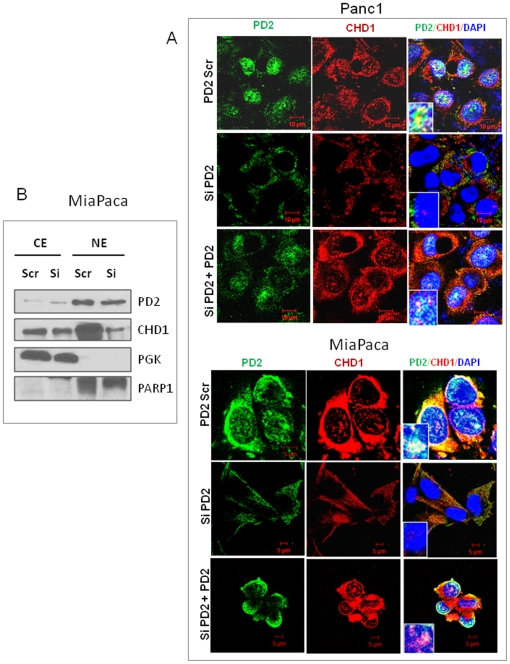
hPaf1/PD2 assists in CHD1 nuclear import in pancreatic cancer cells. (**A**) Panc1 and MiaPaCa cells were transfected with scrambled and PD2 RNAi oligos and 48 hours post transfection immunofluorescence analysis was carried out. Confocal images show that there is decreased localization of CHD1 in the nucleus (blue, stained with DAPI) of PD2 KD pancreatic cancer cells (middle panel) compared to the scrambled cells (top panel). Cells were further retransfected with pBABE-hygro PD2 construct for ectopic expression of PD2, 72 hours post intital transfection. Confocal images illustrate addition of exogenous PD2 reshuttles CHD1 back to the nucleus (bottom panel). The inset represents PD2 and CHD1 localization in the nucleus (stained with DAPI). (**B**) Western blot analysis shows the distribution of CHD1 level in cytoplasmic and nuclear extracts of PD2 knockdown vs. scrambled Panc1 and MiaPaCa cells.

Chromatin remodeling protein CHD1 is known to interact with transcription elongation factors and localize to actively transcribed genes [31]. In yeast, CHD1 functions during transcription elongation through interactions with elongation factors, Spt4-Spt5 and Spt16-Pob3. Interestingly, CHD1 is also found to interact with the Rtf1 subunit of the yeast PAF complex that associates with RNA Polymerase II and regulates transcription elongation [31]. Although the Rtf1 is present in humans, it is not as a part of the hPAF complex. Therefore, we tested the possibility that the hPaf1/PD2 subunit of the human PAF complex interacts with CHD1 using co-immunoprecipitation studies. Western blot analysis of the co-immunoprecipitates shows that PD2 interacts with CHD1 in both cytoplasm as well as nuclear extracts of pancreatic cancer cells, Panc1 and MiaPaCa (**Fig. 4**). We further analyzed the interaction between PD2 and CHD1 by co-localization studies using confocal microscopy (**Fig. 5A**). The CHD1 staining (red) was found to overlap with the PD2 staining (green), both in cytoplasm as well as in the nucleus of pancreatic cancer cells in corroboration with the biochemical data. These results suggest that PD2 and CHD1 interact and further participate in chromatin structure remodeling in pancreatic cancer.

### Nuclear transport of CHD1 is facilitated by hPaf1/PD2 in PC cells

Yeast homologue of CHD1 is found to be localized in the cytoplasm as well as in the nucleus of cells, indicating that it shuttles between the two cellular compartments to carry out its function in determination of chromatin architecture during transcription elongation. Further studies have shown that CHD1 is excluded from chromosomes during mitosis and again reassociates back with the chromatin of the re-forming nucleus during telophase-cytokinesis [29]. Interestingly, recent studies done in our lab have shown that hPaf1/PD2 expression is regulated in a cell-cycle dependent manner in pancreatic cancer cells [32]. More so, the studies suggested that hPaf1/PD2 is shuttled and accumulates in the cytoplasm during mitosis, whereas it appears to concentrate within the condensed chromatin during metaphase [29]. Given that hPaf1/PD2 interacts as well as regulates the level of CHD1 in pancreatic cancer cells, and their similarity in pattern of nuclear-cytoplasmic localization, we tested whether PD2 helps in nuclear transport of CHD1 in pancreatic cancer cells. To this end, we compared the CHD1 distribution in the cytoplasm and nucleus of pancreatic cancer cells between scrambled and PD2 knockdown pancreatic cancer cells using confocal microscopy. The immunofluorescence studies clearly revealed that PD2-specific siRNA treated cells had a reduced level of hPaf1/PD2, which was primarily localized in the cytoplasm compared to scrambled siRNA treated cells. Interestingly, along with reduced PD2 expression, the PD2 knockdown cells showed a decrease in the nuclear localization of CHD1 in both MiaPaCa and Panc1 cells (**Fig. 5A**). Furthermore, Western blot analysis of cytoplasmic and nuclear extracts from scrambled and PD2 knockdown MiaPaCa cells also showed a reduced CHD1 level in the nuclear extracts of PD2 depleted cells (**Fig. 5B**). The suggested role of PD2 in the CHD1 nuclear transport was reconfirmed by a rescue experiment in which the PD2 knockdown cells were further transfected with a pBABE-hygro vector construct expressing full-length PD2. Significantly, re-expression of PD2 in these cells showed a recovery of nuclear localization of CHD1, similar to the scrambled si-RNA treated cells. PD2 and CHD1 were also observed to have a similar distribution pattern in cytoplasm and nuclear extract of other pancreatic cancer cells (**Fig. S2**). Therefore, decreased nuclear accumulation of CHD1 in PD2 knockdown cells indicates that hPaf1/PD2 facilitates the nuclear import of CHD1 in pancreatic cancer cells to carry out its function. This shuttling is hampered in the case of hPaf1/PD2 depleted cells.

### hPaf1/PD2 knockdown affects nucleosomal remodeling in PC cells

**Figure 6:**
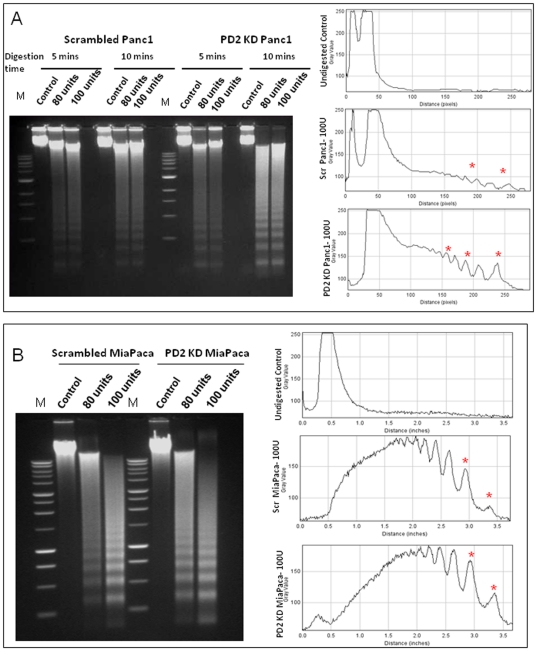
Micrococcal nuclease digestion altered in hPaf1/PD2 knockdown pancreatic cancer cells. The micrococcal nuclease digestion pattern of nuclei isolated from scrambled and PD2 kd Panc1 (**A**) and MiaPaCa cells (**B**). Nuclei were digested with increasing concentrations of micrococcal nuclease enzyme (0.80 and 100 units) for 5 and 10 mins followed by DNA extraction using phenol-chloroform method. Five µg of DNA was then separated in 1.5% agarose gel. The figures on the right hand side represent densitometry analysis of the DNA banding pattern and the asterix in red shows difference between scrambled and knockdown cells.

From recent studies it is becoming apparent that the nuclear architecture and gene positioning contributes highly to gene regulation [33]. The chromatin remodeling enzymes influence this nuclear architecture restructuring by repositioning of the nucleosomes, thereby facilitating the progress of the RNA Polymerase II and associated factors through transcription units. The hPAF complex is known to be required for efficient transcriptional elongation through chromatin. The yeast PAF complex was found to mediate the increased efficiency of transcriptional elongation by promoting nucleosomal destabilization and histone removal [20]. Our data shows that hPaf1/PD2 interacts with and regulates the ATP-dependent chromatin remodeling enzyme, CHD1. Therefore, in this regard, we tested whether knockdown of hPaf1/PD2 alters the nucleosomal positioning in pancreatic cancer cells. To verify this, we transiently knocked down hPaf1/PD2 in pancreatic cancer cells Panc1 and MiaPaCa using the siRNA approach and performed micrococcal nuclease digestion to examine nucleosomal occupancy. The variation in the pattern of the digested DNA ladder is reflective of any change in the nucleosomal occupancy. Comparison of the scrambled and PD2-specific siRNA treated cells showed different micrococcal nuclease digestion patterns in Panc1 (**Fig. 6A**) and MiaPaCa (**Fig. 6B**) cell lines. In both cell types, each of the DNA ladder bands corresponds to either the mononucleosome (147 bp) or polynucleosomes, in successively increasing size. The ladder shows an increase in intensity with increasing concentration of the micrococcal nuclease enzyme, indicating a higher degree of digestion as the ratio of enzyme to DNA ratio increases. Further, the intensity of the DNA ladder is higher in the case of the PD2 knockdown cells compared to the control cells. The densitometric analysis (Fig. 6A,B right panel) shows the difference (marked in red asterix) in intensity of the shortest band corresponding to the mononucleosome between scrambled and knockdown cells. This indicates that the internucleosomal DNA is more accessible for MNase digestion in the case of the knockdown cells. This is representative of the global change in the nucleosomal positioning and hence does not reflect the chromatin remodeling that could account for a change in any particular gene expression. Both the pancreatic cancer cells, Panc1 and MiaPaCa, show a similar variation between control and PD2 knockdown cells. However, the change is more prominent in the case of Panc1 cells which have higher PD2 levels because of the amplification of the 19q13.2 locus. The above observations suggest that PD2 has a direct bearing on the regulatory role of CHD1 in chromatin remodeling, probably by regulating its nuclear import.

## Discussion

Altered gene expression due to changes in the compact chromatin structure and posttranslational histone modifications has often been considered as a significant contributing factor for tumor development [34]. Progression of pancreatic cancer, which has a very poor prognosis and less than a 5% survival rate of five years, is found to be associated with a host of changes in gene expression. Here, we document that inhibition of hPaf1/PD2, a subunit of the human RNA Polymerase II associated complex which is known to be overexpressed in pancreatic cancer, and leads to a reduction in the di and tri-methylation levels of histone H3 lysine 4 residues in pancreatic cancer cells. A decrease in hPaf1/PD2 level also led to a corresponding decrease in the level of MLL1, a histone methyltransferase that regulates H3K4 residue methylation [3]. Inhibition of hPaf1/PD2 also led to a reduction in the level of a chromatin remodeling enzyme CHD1 that specifically binds H3K4Me2/3 and prevents its nuclear import. Furthermore, hPaf1/PD2 knocked down pancreatic cancer cells show an altered pattern of micrococcal nuclease digestion compared to the control cells, indicating that PD2 might play an important role in chromatin structure rearrangement through CHD1 in pancreatic cancer.

We found that a siRNA mediated reduction of hPaf1/PD2 level could decrease the level of di- and tri-methylation of histone H3 at lysine 4 residue, although the change in monomethylation was insignificant in pancreatic cancer cells. However, monomethylation is considered more as a steady state condition, whereas di and tri-methylation of H3 tails on K4 (K4Me3) at the 5′ end of the transcribed region is a signature motif of highly expressed genes and is known to enhance transcriptional elongation. Reduction in hPaf1/PD2 level also led to a decrease in the level of the MLL1 protein, a mammalian homologue of the yeast Set1 histone methyltransferase. Our studies also show that PD2 colocalizes with MLL1 in pancreatic cancer cells. Interestingly, earlier studies suggest that the yeast PAF complex is required for recruitment of the COMPASS methyltransferase to RNA Polymerase II and subunits of this complex interact physically [4]. Further, a recent publication showed that only the hPaf1/PD2 subunit of the human PAF complex interacts with MLL1 [26]. Therefore, our results suggest that hPaf1 may regulate the histone methylation level in pancreatic cancer cells by regulating the expression of HMTs like MLL1, thereby linking transcriptional elongation to chromatin modification. Given that pancreatic cancer is associated with changes in gene expression, these changes in histone methylation marks might contribute to pancreatic cancer development and progression. Our current observations are corroborated by studies done with the yeast PAF complexes, which also play similar roles in transcriptional elongation and regulation of mRNA stability as compared to the human homologue. Studies show that the yPAF complex regulates post-translation histone modifications such as methylation and ubiquitynation. Therefore, it can be speculated that overexpression of hPaf1/PD2 in pancreatic cancer might lead to enhanced histone methylation and upregulation of gene expression indiscriminately. Interestingly, a recent study revealed that MLL1 histone methyltransferase is required for promoter methylation of MDR1 (multi drug resistance gene), a member of the ATP-binding cassette (ABC) transporter family that confers tumor drug resistance by actively effluxing a number of antitumor agents [35]. As evident from the studies done by Milne *et al.* and Muntean *et al.*, PAF1 complex is required for the recruitment of the MLL1 complex at the HOX loci and subsequent transformation [26], [27]. Hence, it might be postulated that the human PAF complex regulates methylation at histone 3 lysine 4 residues through MLL1 and might control gene expression of key proteins involved in tumor initiation, development, progression or resistance to antitumor therapy.

CHD1 is a protein belonging to the family of ATPase dependent chromatin remodelers which specifically bind to di and tri-methylated histone H3 at lysine 4 residue [6]. In this study, we found that along with histone methylation, a reduction in hPaf1/PD2 level also decreased the cellular level of CHD1 protein. CHD1 is localized both in the cytoplasm and the nucleus of cells. Interestingly, PD2 knockdown pancreatic cancer cells seemed to have reduced nuclear localization of CHD1 compared to the scrambled cells, suggesting that the process of CHD1 nuclear shuttling is hampered due to hPaf1 knockdown. Earlier studies have shown that nuclear-cytoplasmic localization of both PD2 and CHD1 are cell-cycle dependent [29], [32]. There is a spatial-temporal correlation between the distribution of PD2 and CHD1 in cells. CHD1 is absent from chromosomes during mitosis and reassociates back with the nucleus during cytokinesis. Strikingly, hPaf1 is also degraded during mitosis whereas it drives cell-cycle progression in the G1, S and G2 phase by regulating cyclin B1, D1 and E1 transcription initiation. The earlier studies and the current results indicate that PD2 is perhaps involved in facilitating the nuclear import of CHD1 to the nucleus, wherein it helps in remodeling of the chromatin structure. Moreover, the secondary structure of hPaf1/PD2 consists of a RCC1 (Regulator of chromatin condensation) domain [19] which is homologous to the RCC1 domain present in RanGTPase, a protein involved in nuclear transport [36]. The potential involvement of the PD2 RCC1 domain in nuclear transport of CHD1 needs further investigation. In this regard, a recent perspective on the stem cell property of self renewal and pluripotency emphasizes that CHD1 is essential for open chromatin and pluripotency of embryonic stem cells, and for somatic cell reprogramming to the pluripotent state [37]. Interestingly, a recent study from our lab demonstrates that the Paf1 subunit interacts with Oct3/4 and is also required for the maintenance of mouse embryonic stem cell self renewal and pluripotency [22]. Our results also show that PD2 interacts with CHD1 both in cytoplasm as well as in the nucleus of pancreatic cancer cells. It has been proposed that this interaction might be important in stabilization of the binding of CHD1 to methylated histones as well as in facilitation of its nuclear-cytoplasmic shuttling. Overall, these studies strongly suggest a concerted function between Paf1 and CHD1 proteins in regulating gene expression, thereby affecting the process of cell cycle progression, differentiation or tumor development.

**Figure 7:**
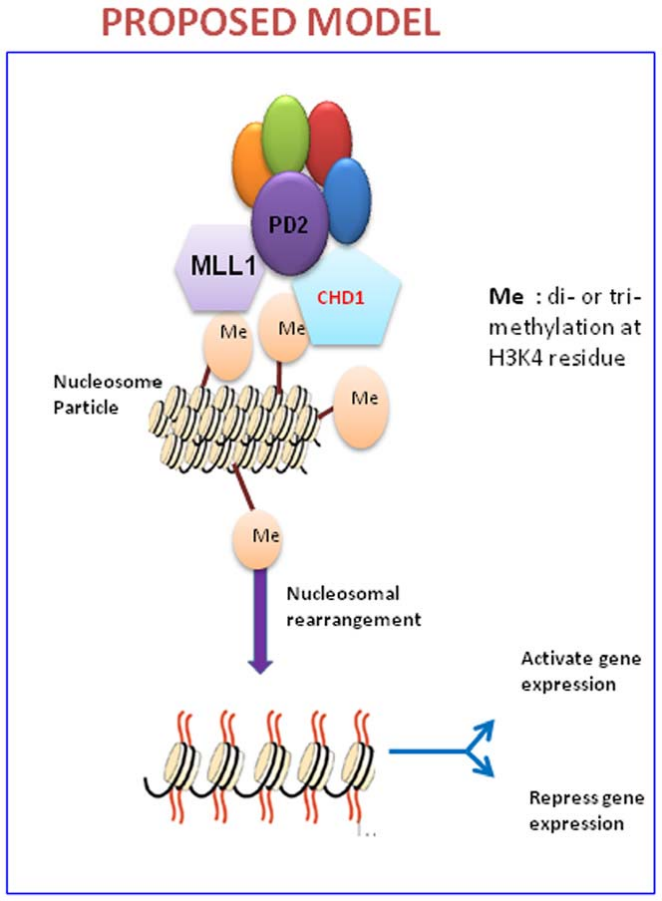
Proposed model for regulation of histone methylation and chromatin remodeling by hPaf1/PD2. PD2 affects methylation at H3K4 residue by regulating the histone methyltransferase MLL1. CHD1, a chromatin remodeling protein, that interacts with PD2, then binds to these di-methylated and trimethylated H3K4 moieties. The expression as well as nuclear transport of the CHD1 protein is also regulated by hPaf1/PD2. Binding of CHD1 to the modified histones finally leads to remodeling of the nucleosomal structure resulting in a change in gene expression.

Chromatin assembly may occur by an active histone deposition mechanism in which CHD1 function is involved in the transfer of histones to DNA as well as the formation of periodic arrays of nucleosomes [5]. Thus, molecular motor proteins, such as CHD1, function not only in the remodeling of existing nucleosomes but also in de novo nucleosome assembly from DNA and histones. The chromatin is always in a dynamic state during gene expression; the histones are constantly displaced and reintroduced to facilitate transcription. These processes are carried out by active chromatin remodeling proteins like CHD1 by harnessing the ATP energy. From the micrococcal nuclease digestion experiment, it was observed that the PD2 knockdown cells show a more intense DNA laddering compared to control cells, indicating an increased susceptibility to enzyme digestion. Since PD2 regulates the active transcription mark, H3K4 di and trimethylation, the knockdown of hPaf1/PD2 should lead to a decreased transcription. Apparently, the micrococcal nuclease digestion pattern seems contradictory, since the PD2 knockdown looks more accessible to digestion, indicative of open chromatin and active transcription. However, based on the dynamic model of nucleosomal assembly and disassembly, it may be suggested that the hPaf1/PD2 knockdown cells are in a more or less steady state due to decreased CHD1 levels and hence show a specific digestion pattern. On the other hand, the cancer cells with an overexpression of hPaf1/PD2 and CHD1 have active transcriptional upregulation and, as a result, chromatin rearrangement occurs, constantly making the chromatin template less accessible to nuclease digestion. However, this study represents a global change in the nucleosomal arrangement, although any change in chromosomal structure leading to expression or repression of specific genes requires a more in-depth analysis of the nucleosomal structure using specific primers corresponding to the promoter region of target genes for PD2 and CHD1. Therefore, our results suggest that the hPaf1/PD2 subunit of the human PAF complex interacts with the chromatin remodeling protein CHD1 in pancreatic cancer cells, facilitating its nuclear import and thereby regulating its function of nucleosomal remodeling (**Fig. 7**). Future studies may address the genes whose expression is controlled by such an interaction between hPaf1 and CHD1 and its significance in pancreatic cancer development and progression. Overall, this study emphasizes the role of key transcriptional regulators, like the hPAF complex, in epigenetic changes and subsequent variation of the chromosomal architecture, thereby determining the state of gene expression critical to the process of tumorogenesis.

## Supporting Information

Figure S1 **Quantitative Real-time PCR analysis of PD2 and CHD1 mRNA expression in hPaf1 knockdown vs scrambled Panc1 cells.** (**A**) Quantitative real-time PCR analysis for mRNA expression of PD2 and CHD1 was done using the following primers- CHD1 FP 5′-TGAGCCATTTCTGTTACGCCGAGT-3′, CHD1 RP 5′-TGAGGTACTGCCCTTGGAACCTTT-3′. Results show reduced level of PD2 mRNA in Panc1 cells transfected with PD2 siRNA as compared to scrambled siRNA treated cells. CHD1 mRNA level also shows a corresponding decrease in expression in PD2 knockdown cells compared to control cells. The mRNA expression level was determined after normalization with β-actin mRNA expression. (**B**) Western blot analysis of PD2 and CHD1 in PD2 overexpressed HPAF/CD18 pancreatic cancer cells. hPaf1/PD2 was ectopically overexpressed in HPAF/CD18 pancreatic cancer cells having low endogenous level of PD2 by transfection with pBABE.hygro vector containing full-length PD2 construct. Western blot analysis shows that along with increase in PD2 expression, CHD1 protein level as well as the histone H3 lysine 4 tri-methylation mark is also increased in the PD2 overexpressing HPAF/CD18 cells compared to vector transfected cells. β-actin is used as the loading control.
(TIF)

Figure S2 **Cytoplasmic and nuclear distribution of PD2 and CHD1 in different pancreatic cancer cell lines.** Immunoblotting with cytoplasmic and nuclear extracts collected from pancreatic cancer cell lines – Capan2, S2CP9 and BxPC3 show that there is a correlation between the distribution of PD2 and CHD1 in the cellular compartments. Capan2 has higher PD2 as well as CHD1 content in the cytoplasmic extract whereas S2CP9 and BxPC3 have higher level of both PD2 and CHD1 in the nuclear extract. PGK and PARP serve as the loading controls for cytoplasmic and nuclear extracts respectively.
(TIF)

Figure S3 **Downregulation of hPaf1/PD2 using different pool of siRNA and its effect on PAF complex subunits, H3K4 methylation, MLL1 and CHD1 level.** Other than the siRNA (Dharnacon) used for knockdown of PD2/hPaf1, we also used a different pool of siRNAs, obtained from SantaCruz Biotechnologies (sc-76034) to downregulate PD2 in Panc1 and MiaPaCa pancreatic cancer cells. The effect of PD2 knockdown using the new pool of siRNAs was investigated by analyzing the protein levels of other PAF complex subunits, H3K4 methylation, histone methyltransferase MLL1 and chromatin remodeling protein CHD1 by western blotting. The bar diagram represents quantification of the western blot figures. β-actin is used as the loading control and for normalization of the quantification data.
(TIF)
