# Supplementary figures and images for: Human RNA Polymerase II-Association Factor 1 (hPaf1/PD2) Regulates Histone Methylation and Chromatin Remodeling in Pancreatic Cancer
Source: PLoS One. 2011 Oct 27;6(10):e26926. doi: 10.1371/journal.pone.0026926 (PMC3203178; doi:10.1371/journal.pone.0026926)

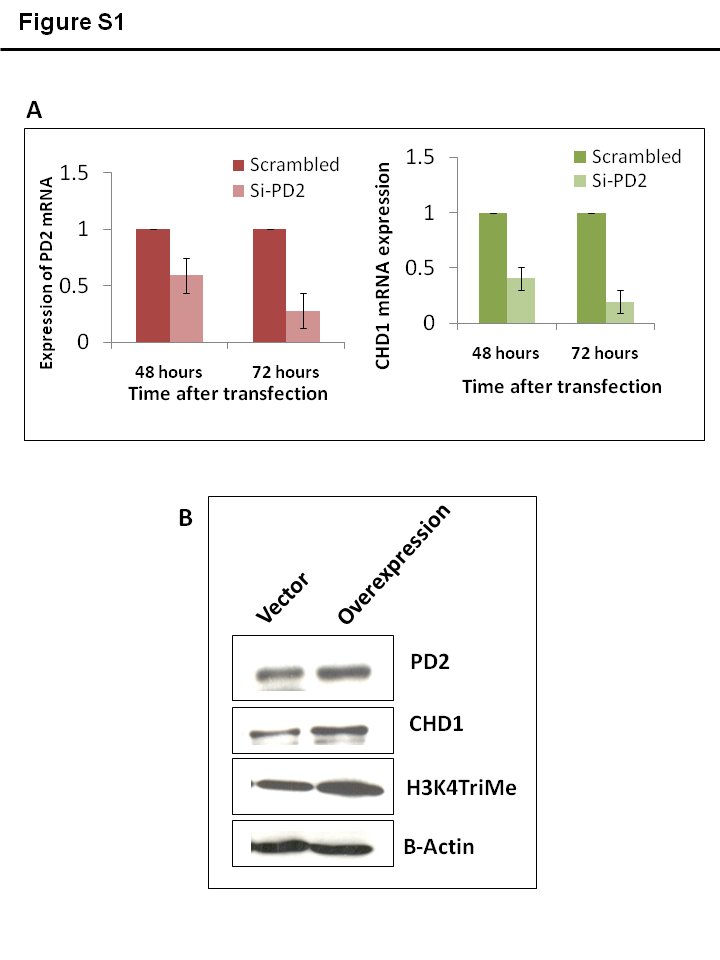

Supplement: Figure S1 — Quantitative Real-time PCR analysis of PD2 and CHD1 mRNA expression in hPaf1 knockdown vs scrambled Panc1 cells. (A) Quantitative real-time PCR analysis for mRNA expression of PD2 and CHD1 was done using the following primers- CHD1 FP 5′-TGAGCCATTTCTGTTACGCCGAGT-3′, CHD1 RP 5′-TGAGGTACTGCCCTTGGAACCTTT-3′. Results show reduced level of PD2 mRNA in Panc1 cells transfected with PD2 siRNA as compared to scrambled siRNA treated cells. CHD1 mRNA level also shows a corresponding decrease in expression in PD2 knockdown cells compared to control cells. The mRNA expression level was determined after normalization with β-actin mRNA expression. (B) Western blot analysis of PD2 and CHD1 in PD2 overexpressed HPAF/CD18 pancreatic cancer cells. hPaf1/PD2 was ectopically overexpressed in HPAF/CD18 pancreatic cancer cells having low endogenous level of PD2 by transfection with pBABE.hygro vector containing full-length PD2 construct. Western blot analysis shows that along with increase in PD2 expression, CHD1 protein level as well as the histone H3 lysine 4 tri-methylation mark is also increased in the PD2 overexpressing HPAF/CD18 cells compared to vector transfected cells. β-actin is used as the loading control. (TIF) [file pone.0026926.s001.tif]

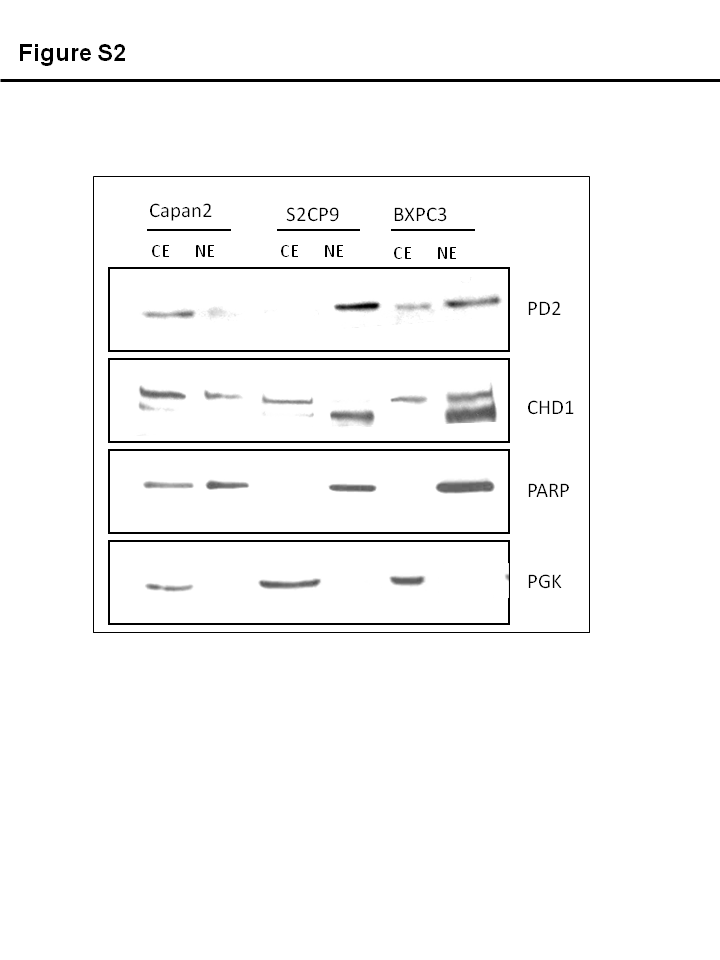

Supplement: Figure S2 — Cytoplasmic and nuclear distribution of PD2 and CHD1 in different pancreatic cancer cell lines. Immunoblotting with cytoplasmic and nuclear extracts collected from pancreatic cancer cell lines – Capan2, S2CP9 and BxPC3 show that there is a correlation between the distribution of PD2 and CHD1 in the cellular compartments. Capan2 has higher PD2 as well as CHD1 content in the cytoplasmic extract whereas S2CP9 and BxPC3 have higher level of both PD2 and CHD1 in the nuclear extract. PGK and PARP serve as the loading controls for cytoplasmic and nuclear extracts respectively. (TIF) [file pone.0026926.s002.tif]

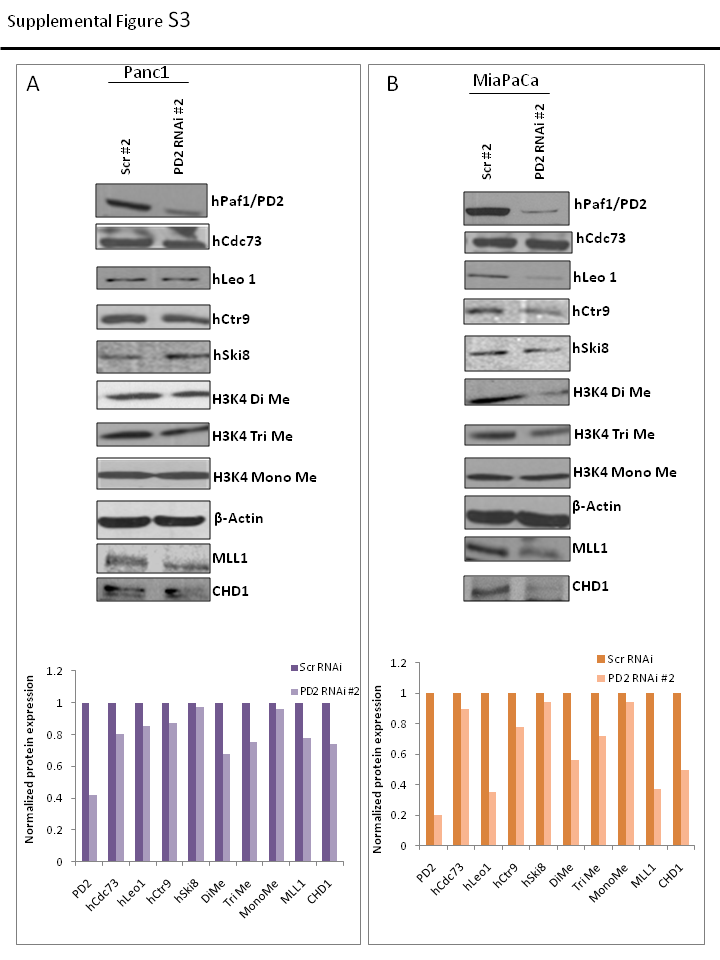

Supplement: Figure S3 — Downregulation of hPaf1/PD2 using different pool of siRNA and its effect on PAF complex subunits, H3K4 methylation, MLL1 and CHD1 level. Other than the siRNA (Dharnacon) used for knockdown of PD2/hPaf1, we also used a different pool of siRNAs, obtained from SantaCruz Biotechnologies (sc-76034) to downregulate PD2 in Panc1 and MiaPaCa pancreatic cancer cells. The effect of PD2 knockdown using the new pool of siRNAs was investigated by analyzing the protein levels of other PAF complex subunits, H3K4 methylation, histone methyltransferase MLL1 and chromatin remodeling protein CHD1 by western blotting. The bar diagram represents quantification of the western blot figures. β-actin is used as the loading control and for normalization of the quantification data. (TIF) [file pone.0026926.s003.tif]
